# Analysis of microbial community evolution, autolysis phenomena, and energy metabolism pathways in *Pholiota nameko* endophytes

**DOI:** 10.3389/fmicb.2024.1319886

**Published:** 2024-04-16

**Authors:** Huan Zhao, Yan He, Yichu Wang, Xiaolong He, Ruihua Zhao, Bo Liu

**Affiliations:** College of Life Sciences, Yan’an University, Yan’an, China

**Keywords:** *Pholiota nameko*, microbial diversity, community succession, autolysis, energy metabolism

## Abstract

**Introduction:**

Pholiota nameko is a widely consumed edible fungus. This study focuses on two crucial developmental stages of Pholiota nameko, namely, mycelium and ascospores. The objectives of this research were to investigate changes in microbial diversity and community structure during the growth of Pholiota nameko and to analyze the adaptability of the dominant strains to their respective habitats through metabolic.

**Methods:**

Specifically, we conducted second-generation sequencing of the 16S rRNA gene (Illumina) on samples obtained from these stages. In addition, we isolated and characterized endophytes present in Pholiota nameko, focusing on examining the impact of dominant endophyte genera on autolysis. We also conducted a metabolic pathway analysis.

**Results and discussion:**

The results unveiled 578,414 valid sequences of Pholiota nameko endophytic fungi. At the phylum level, the dominant taxa were Basidiomycota, Ascomycota, Zoopagomycota, and Mucoromycota. At the genus level, the dominant taxa observed were Pholiota, Inocybe, Fusarium, and Hortiboletus. For endophytic bacteria, we obtained 458,475 valid sequences. The dominant phyla were Proteobacteria, TM6, Firmicutes, and Bacteroidetes, while the dominant genera were Edaphobacter, Xanthomonas, Burkholderia, and Pseudomonas. Moreover, we identified the isolated strains in Pholiota nameko using 16S rDNA, and most of them were found to belong to the genus Pseudomonas, with Pseudomonas putida being the most prevalent strain. The findings revealed that the Pseudomonas putida strain has the ability to slow down the breakdown of soluble proteins and partially suppress the metabolic processes that generate superoxide anion radicals in Pholiota nameko, thereby reducing autolysis. Additionally, our results demonstrated that molybdenum enzyme-mediated anaerobic oxidative phosphorylation reactions were the primary energy metabolism pathway in the Pseudomonas putida strain. This suggests that the molybdenum cofactor synthesis pathway might be the main mechanism through which Pholiota nameko adapts to its complex and diverse habitats.

## Introduction

*Pholiota nameko*, commonly known as *pearl oyster mushroom*, *slider mushroom*, and *glossy cap scale umbrella*, is a large edible fungus. It belongs to the non-septate stramenopiles subclass, the Umbelliferae order, and the Umbelliferae family (Yang et al., 2019). *Pholiota nameko* is not only highly delicious and nutritious, but it also contains polysaccharide compounds that exhibit tumor growth inhibition and provide protection against infections caused by staphylococcus, *Escherichia coli*, pneumococcus, and *Mycobacterium tuberculosis* (Abreu et al., 2021). It is abundant in crude protein, fat, carbohydrates, crude fibers, ash, calcium, phosphorus, iron, vitamin B (VB), vitamin (VC), niacin, and other essential amino acids, all of which contribute positively to human health (Kawamura et al., 2007). Furthermore, *Pholiota nameko* ranks among the top 10 globally traded mushrooms and has been recommended by the Food and Agriculture Organization for cultivation in underdeveloped nations (Shao et al., 2022). With a history spanning over 6,000 years, *Pholiota nameko* has gained immense popularity as both a food and a medicinal mushroom. Additionally, research suggests that *Pholiota nameko* has prebiotic properties, influencing the diversity and relative abundance of gut flora (Zhang et al., 2022).

Endophytes are a significant group of microorganisms that play a crucial role in promoting the growth and wellbeing of individual organisms. They also hold potential applications in agriculture and medicine. In the case of *Pholiota nameko*, the development of this species is influenced by changes in the microbial population of its endophytes, which, in turn, affect the production of active substances (Santoyo et al., 2016). Therefore, it is pivotal to explore the microbial communities within *Pholiota nameko* endophytes using second-generation sequencing technology that targets the 16S rRNA gene. This exploration is vital for effectively harnessing the resources of *Pholiota nameko* and facilitating the commercialization of its endophytes. Autolysis, a natural degradation process, occurs in bacteria when they lack external nutrients or perceive an unfavorable external environment for their growth (Sami et al., 2021). However, the *Pholiota nameko* tissue is highly susceptible to autolysis due to its sensitivity, high water content, and lack of protective structures. This vulnerability makes it prone to disease infiltration and mechanical damage, resulting in autolysis and a significant reduction in the utility of *Pholiota nameko* (Ji et al., 2012). Research conducted on *Agaricus bisporus* has suggested a correlation between the storage environment and autolysis, with longer storage periods accelerating the autolysis process. These studies have also indicated a positive relationship between the cell permeability of *Agaricus bisporus* and the duration of storage (Zhu et al., 2019). Researchers have also found that microorganisms accelerate autolysis in *Agrocybe aegerita* by utilizing the nutrients released from the autolyzed substrate for their multiplication (Rodrigues et al., 2017). However, the influence of endophytic bacteria on the autolysis ability of *Pholiota nameko* remains undocumented.

**Figure 1:**
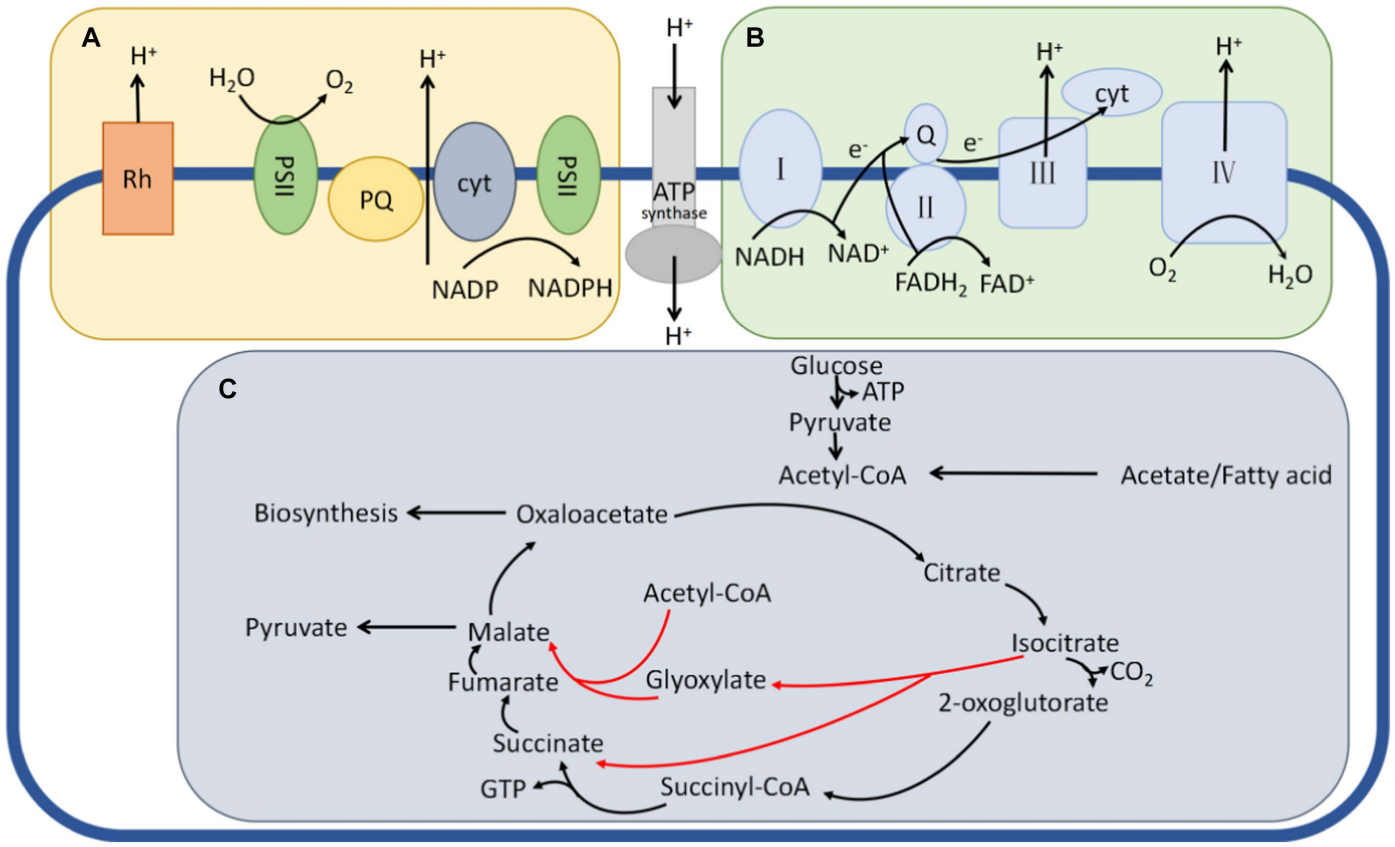
Three typical pathways for energy metabolism in bacteria. **(A)** The phototrophic metabolism route, in which the right pattern relies on chlorophyll and the left pattern relies on retinal plasma. A proton motive force is produced when light pushes protons across the cytoplasmic membrane. This force is employed by ATP synthase, flagellar rotation, and other cellular functions. **(B)** The oxidative phosphorylation of the respiratory electron transport chain, which also produces a proton motive force. **(C)** A set of TCA cycle events that encourage substrate phosphorylation. Enzymes immediately add phosphate groups to ADP during substrate metabolism to create ATP. Isocitric acid produces a glyoxylate shunt (red arrows) to boost carbon–biomass conversion when cellular energy reaches a particular point but instead releases two carbons in the form of CO_2_ when this happens.

Figure 1 shows three distinct energy metabolism pathways in microorganisms: photoenergetic feeding, oxidative phosphorylation, and substrate-level phosphorylation (Kirchman and Hanson, 2013). Photoenergetic nutrition harnesses light as an energy source by utilizing photosynthetic pigments such as chlorophyll, bacteriorhodopsin, or retinal, which generate a proton gradient across the membrane through proton transport. This process facilitates ATP production. Oxidative phosphorylation can be classified into two types depending on the terminal electron acceptor: aerobic respiration, which exclusively uses oxygen as the final electron acceptor in an aerobic environment, and anaerobic respiration, which employs various organic or inorganic compounds as final electron acceptors under anoxic conditions (Tyrrell and Pereira, 1980). Conversely, substrate-level phosphorylation directly transfers high-energy phosphate groups generated during substrate metabolism to adenosine diphosphate (ADP) [or guanosine diphosphate (GDP)], resulting in the synthesis of adenosine triphosphate (ATP) [or guanosine triphosphate (GTP)] (Fernie et al., 2004). In this research, we specifically examined the energy metabolism pathway of anaerobic respiration to investigate the role of energy metabolism in the environmental adaptation of the genus *Pseudomonas putida*.

The objectives of this study were multifaceted. First, our goal was to identify the successional changes in the microbial community during the growth of *Pholiota nameko*. Second, we aimed to investigate the influence of the dominant strain of *Pholiota nameko* on its autolysis. Additionally, we aimed to conduct targeted studies focusing on crucial enzymes or proteins involved in bacterial energy metabolism. Through these investigations, we sought to establish the presence and distribution of anaerobic respiratory energy metabolism pathways in the dominant strain of *Pholiota nameko*.

## Materials and methods

### DNA extraction and high-throughput sequencing

The *Pholiota nameko* samples utilized in this study were provided by the Laboratory of Food and Medicinal Mushrooms, College of Life Sciences, Yan’an University. The samples were inoculated on 18 May 2023 and allowed to reach the mycelial stage. They were then collected on May 28 and further cultivated until the ascospore stage, at which point they were collected on June 18 (Hansen et al., 2020). Before the analysis, the surfaces of the samples were thoroughly sterilized. Subsequently, each sample was placed in 5 mL of sterilized EP solution, flash-frozen using liquid nitrogen for 3 min, and stored at −80°C in a refrigerator.

The genomic DNA was extracted from the samples using the CTAB method, and its purity and concentration were assessed through 1% agarose gel electrophoresis. A suitable amount of the DNA material was then diluted to a concentration of 1 ng/μl using sterile water. Polymerase chain reaction (PCR) amplification of the V3 + V4 variable region was carried out using the primers 341F (5′-CCTAYGGGRBGCASCAG-3′) and 806R (5′-GGACTACNNGGGGTATCTAAT-3′) (Bolyen et al., 2019). The DNA of each sample was extracted under sterile conditions according to the steps provided in the kit using the Fungal Genomic DNA Kit (Tiangen Biochemical Technology Co., Ltd., Cat. No. DP812); the concentration and purity of the sample DNA were detected by agarose gel electroswimming at 1.0%; and PCR amplification was carried out using the primers ITS1F (5′-CTTGGTCATTTAGAGGAAGTAA-3′) and ITS2R (5′-GCTGCGTTCTTCATCGATGC-3′) – GCTGCGTTCTTCATCGATGC-3′ primers for PCR amplification. The target bands were recovered using the Universal DNA Purification and Recovery Kit (TIANGEN, China). For library construction, the NEB Next^®^ Ultra DNA Library Prep Kit was used, and the library was quantified and detected using an Agilent 5,400. Once the library passed the qualification criteria, it was sequenced using a NovaSeq 6,000 (Wang et al., 2019). For analysis, the “Atacama soil microbiome tutorial” from the Qiime2 documentation^1^ was followed. The raw sequence fastq files were imported into a format compatible with QIIME2’s future processing using the qiime tools import plug-in. The QIIME2 dada2 plug-in was then used for quality assurance, trimming, denoising, splicing, and chimera removal, resulting in the final feature sequence table (Callahan et al., 2016). To assign taxonomic information, the representative sequences of the ASVs were compared to the pre-trained GREENGENES database version 13_8 with a 99% similarity threshold using the QIIME2 feature-classifier plugin. The database was pruned to include only the V3V4 region targeted by the 341F/806R primer pairs. This comparison allowed the generation of a taxonomic information table at the species level (Bokulich et al., 2018).

### Isolation and identification of endophytic bacteria

Fresh *Pholiota nameko* mushrooms were immersed in sterile water for 5 min within a two-person ultra-cleaning workbench. After removing them, excess surface moisture was blotted out using filter paper. Subsequently, they were immersed in 75% ethanol for 3 min and rinsed four times with sterile water. Next, they underwent a 3 min treatment with 3% sodium hypochlorite, and the surface moisture was again blotted out using filter paper. The mushrooms were then set aside for further use. The epidermis of the disinfected *Pholiota nameko* was removed using the grinding method in the two-person purification workbench. The internal tissues of the mushrooms were transferred into a mortar, and sterile water was added for grinding. After an appropriate incubation period, the supernatant was spread onto an NA medium plate. The control group underwent a final round of washing and was then incubated at a constant temperature of 37°C. The strains that grew after the plating process were isolated using the plate delineation method. Single colonies that were separated were gently removed from the nutrient agar (NA) plate using an inoculating ring and transferred to NA for isolation culture. The plates were then placed in a constant temperature incubator at 37°C for 24 h. Afterward, the isolated and purified strains were inoculated into a liquid medium using an inoculation loop and shaken for 12 h at 37°C in a constant temperature incubator. The resulting culture was then subjected to a centrifugation process at 10,000 g for 15 min at 4°C. The supernatant was carefully discarded, and this centrifugation step was repeated. The supernatant was poured off again, leaving behind the precipitate for identification. DNA was extracted using the CTAB method (Murray and Thompson, 1980). Shanghai Bioengineering Company sequenced the collected DNA, and the Basic Local Alignment Search Tool (BLAST) was used to compare the sequencing results. Using MEGA 6.0’s Neighbor-Joining (NJ) function, the evolutionary tree was built (Crisafi et al., 2011; Bella et al., 2013).

### *Pholiota nameko* evaluation of autolysis indicators for dominant genera

*Pholiota nameko* were stored at room temperature (25°C) for 1 week, with samples randomly selected at daily intervals. A 25 g sample was obtained using a sterilized sampler and mixed with 225 mL of sterilized 0.85% saline solution. Following Koch’s postulates, isolated and purified strains were prepared as bacterial suspensions using sterile water. These suspensions were then inoculated onto previously sterilized *Pholiota nameko* using a spraying method with ultraviolet radiation. Each strain was inoculated into three boxes, with each box containing six *Pholiota nameko*. The inoculated *Pholiota nameko* were exposed to *Pseudomonas* BAS and incubated at 37°C and 28°C, while control samples were observed once daily in a sterile room maintained at 25°C.

Five distinct classes were used to grade the autolysis index, and Table 1 displays the grading standards (Zhu et al., 2007). The autolysis index of Pholiota nameko was determined using the following formula:


$$\text{Autolysis}\;\text{index}=\sum \left[\begin{array}{l} \text{Pholiota}\;\text{nameko}\;\text{autolytic}\;\text{grade}\\ \times \left(\frac{\text{Number}\;\text{at}\;\text{this}\;\text{level}}{\text{aggregate}}\right) \end{array}\right]$$

**Table 1:** *Pholiota nameko* autolysis index grading criteria.

Quality level	Description of *Pholiota nameko* status
Level 0	Fresh and intact, no open umbrellas
Level 1	Slightly open umbrella, no discoloration of the cap, slight discoloration of the stipe, and a brown area less than 1/4 of the whole mushroom body
Level 2	Mushroom body 1/2–3/4 open, cap slightly discolored, stipe brown deepened, brown area less than 1/4–1/2 of the whole mushroom body
Level 3	Open more than 3/4 of the umbrella; the cap and stipe are brown and deepened; and the brown area is less than 1/2–3/4 of the whole mushroom body
Level 4	Fully open, cap and stipe completely brown, surface of the mushroom body rotting, with black sap outflow

The Malondialdehyde (MDA) content was determined using the colorimetric method of thiobarbituric acid (Hildebrandt et al., 2008), while the O_2_^−^ content was measured using the method described by Abreu et al. (2021). The soluble protein content was determined using the method developed by Marion (1976). The Catalase (CAT) activity was assessed following the method proposed by Abreu et al. (2021). The Superoxide Dismutase (SOD) activity was measured using the Peroxidase (POD) Vitality Assay Kit (Kawamura et al., 2007), and the POD activity was determined using the guaiacol method (Shah and Nahakpam, 2012).

### Analysis of energy metabolism pathways in Pholiota nameko dominant genera

#### Genome acquisition and evolutionary tree construction

A total of 345 genomic files (.fna files) of dominant *Pholiota nameko strains* were obtained from the National Center for Biotechnology Information (NCBI) database at https://www.ncbi.nlm.nih.gov/. The quality of these genomes was assessed using CheckM (v1.0.12), resulting in 338 valid data files with >95% genome integrity and <5% contamination (Parks et al., 2015). Next, the 338 strains of dominant *Pholiota nameko* were assigned to their respective species using the GTDB-Tk-1.7.0 program (Chaumeil et al., 2020). Furthermore, their average nucleotide identity was calculated using FastANIv1.33, with the fragLen1000 parameter and default settings for other parameters (Jain et al., 2018). A 95% similarity threshold was used to classify them into distinct species, which was complementary to the results obtained from GTDB classification. The comparison files were imported into FastTree (version 2.1.11), and the maximum-likelihood (ML) method with default parameters was used to construct the evolutionary tree (Carmel et al., 2009; Price et al., 2010). As outgroups, Citromicrobium sp. JLT1363 and *Gillisia limnaea* were included in the analysis. To annotate species categorization and strain origin information, the resulting evolutionary tree was visualized with distinct colors using the iTOL Evolutionary Tree Online Modification website^2^ (Kumar et al., 2021).

#### Construction of protein phylogenetic tree

To annotate the molybdenum cofactor synthesis protein gene, the genome annotation file (.gff file) and protein sequence file (.faa file) for the 338 strains were downloaded from the NCBI database. A search was conducted using key phrases, such as “MoaA”-“MoaE,” “MoeA,” and “MogA,” to identify the associated proteins, indicating the presence of related genes in the genome. By locating these proteins in the files using the designated keywords, the existence of the corresponding genes in the genome was confirmed. The genomic evolutionary tree was created using iTOL software to identify the strains possessing the genes responsible for molybdenum cofactor production protein. Additionally, the molybdenum cofactor synthesis protein (MoA) sequences were compared using the MEGAX program, and a phylogenetic tree was generated (Afzal et al., 2019). The raw data were labeled, and the phylogenetic tree was subsequently updated using iTOL.

## Results

### Microbial diversity during *Pholiota nameko* growth

**Figure 2:**
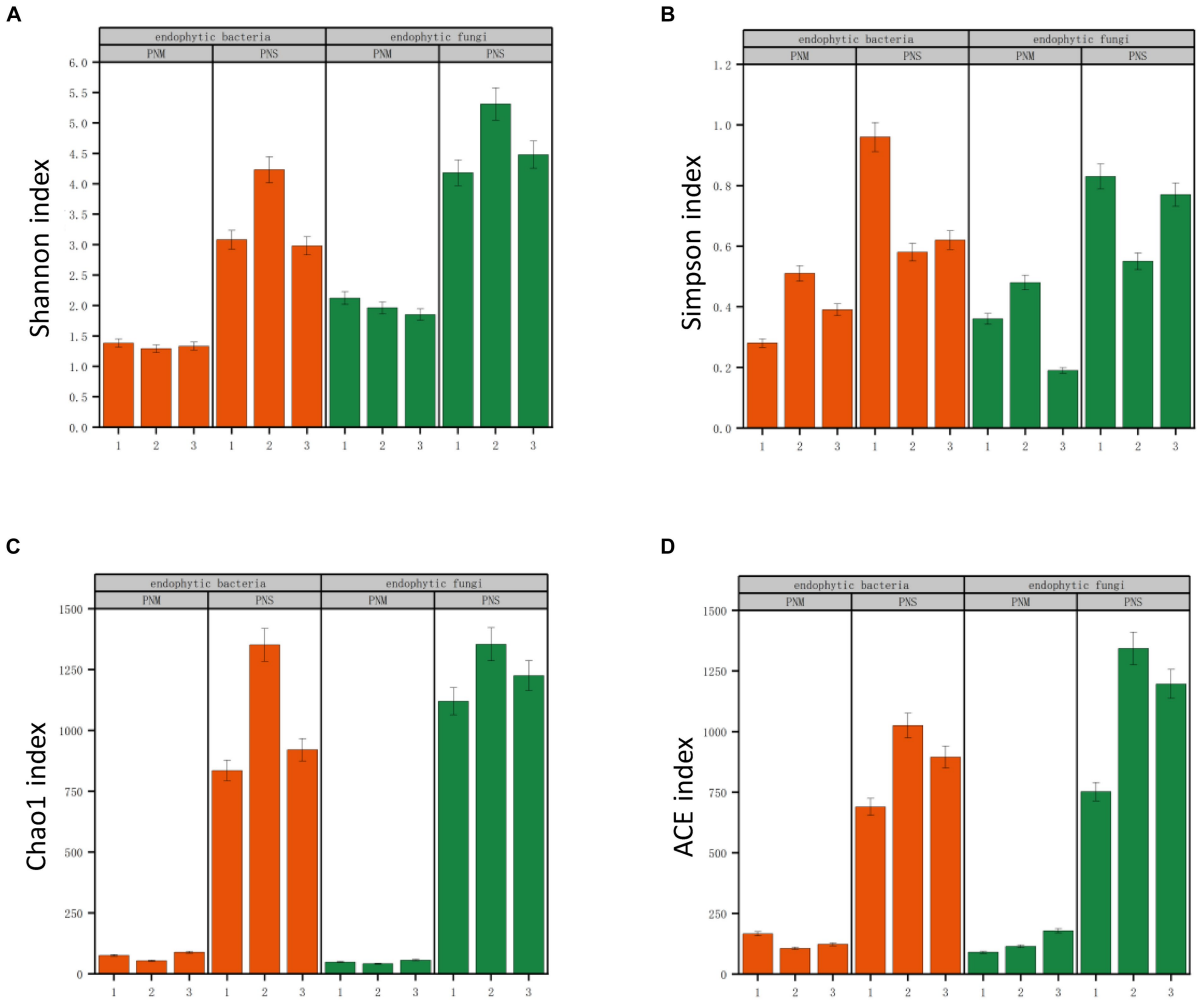
Richness and diversity of microbial communities at different stages of *Pholiota nameko*. **(A)** Shannon index; **(B)** Simpson index; **(C)** Chao1 index; **(D)** ACE index. Lowercase letters above the bars indicate significant differences (*p* < 0.5) between the different growth stages of *Pholiota nameko* under the two subgroups.

A total of 578,414 original sequences were obtained through Illumina NovaSeq sequencing of endophytic fungi. After applying fit filtering and quality screening, 540,337 valid sequences remained. Additionally, 426,036 valid reads were obtained for the endophytic bacteria. The dilution curves for all samples showed an initial increase followed by a gradual leveling off with increasing sequencing depth, indicating an adequate sample size and reliable sequencing data that can be utilized for further investigation (Supplementary Tables S1, S2; Supplementary Figures S1, S2). The Chao 1 and ACE indices were used to assess the biological richness in the community, while the Simpson and Shannon indices were employed to describe species evenness and diversity (Figure 2). Regardless of the developmental stage, the mycelium of *Pholiota nameko* displayed higher richness and diversity in the endophyte community compared to the substrate. These results indicated that the mycelium harbored a more complex and abundant microbial species population.

### Analysis of endophytes community structure

**Figure 3:**
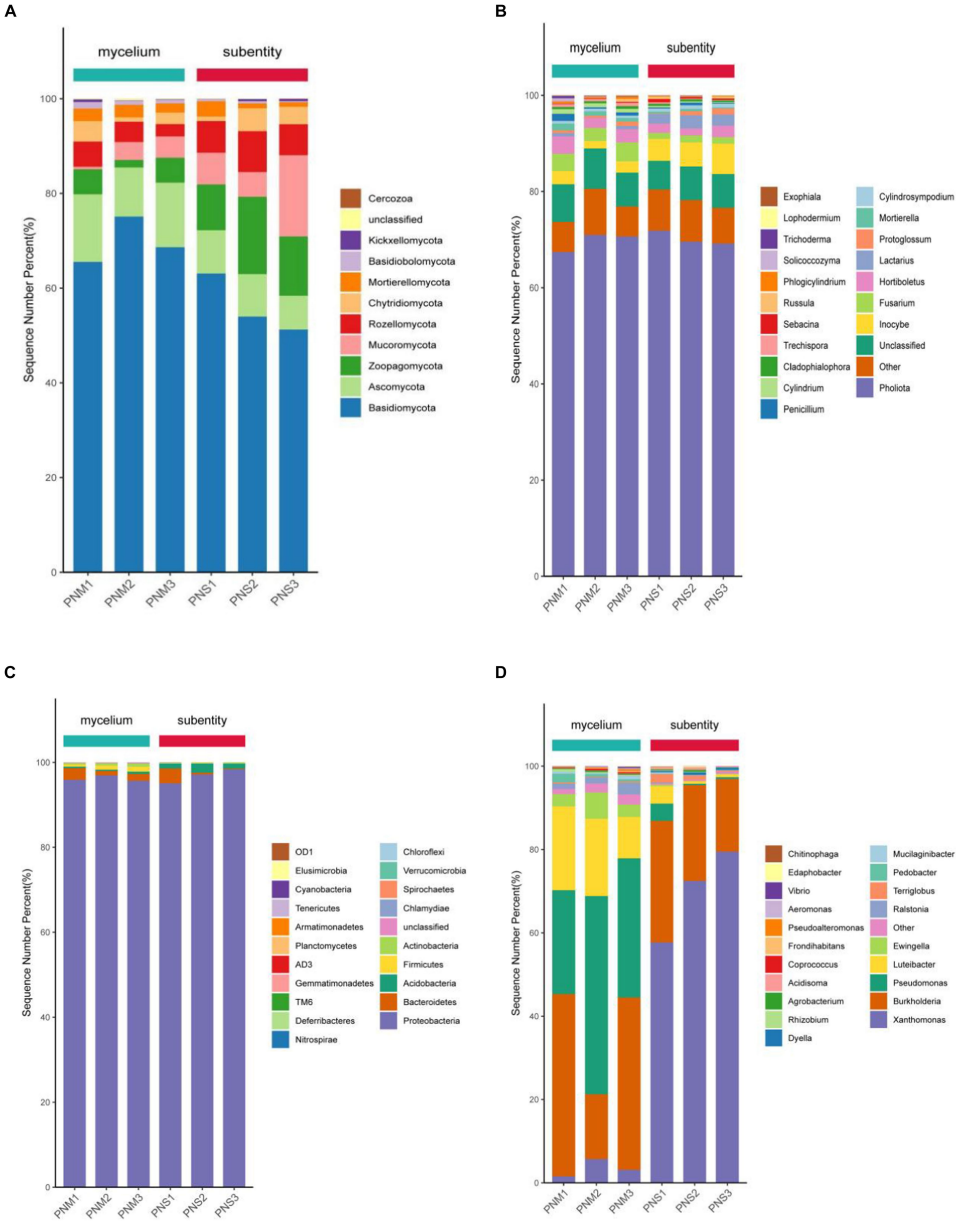
Community composition of microorganisms (top 20 in relative abundance) in different growth stages of *Pholiota nameko*. **(A)** Community composition of endophytic fungi at the phylum level; **(B)** community composition of endophytic fungi at the genus level; **(C)** community composition of endophytic bacteria at the phylum level; **(D)** community composition of endophytic bacteria at the genus level.

The endophytic bacterial community of *Pholiota nameko* was dominated by *Proteobacteria*, *TM6*, *Firmicutes*, and *Bacteroidetes* at the phylum level. Throughout the growth stages of *Pholiota nameko*, there was a decrease in the relative abundance of *Firmicutes* from 2.57 to 0.87%, while *TM6* increased from 1.07 to 3.14%. *Proteobacteria* had a consistently high relative abundance of 95.65 to 98.32%, establishing their complete dominance. At the genus level, *Edaphobacter*, *Xanthomonas*, *Burkholderia*, and *Pseudomonas* were the dominant genera. Specifically, the relative abundance of *Luteibacter* decreased from 25.24 to 18.31% during the growth of *Pholiota nameko* from mycelium to fruiting bodies. Moreover, the relative abundance of *Pseudomonas* decreased from 39.58 to 3.57%, while the relative abundance of *Xanthomonas* increased from 4.27 to 68.53%. Following the transition to a substrate, the relative abundance of *Basidiomycota* decreased from 67.42 to 59.27%, and that of *Ascomycota* decreased from 18.54 to 11.08%. On the other hand, the relative abundance of *Zoopagomycota* increased from 6.71 to 14.93%, and that of *Mucoromycota* increased from 4.88 to 9.64%. These phyla dominated the endophytic fungal community of *Pholiota nameko*. At the genus level, *Pholiota*, *Unclassified*, *Inocybe*, *Fusarium*, and *Hortiboletus* were the dominant genera. Notably, *Pholiota* was the dominant genus in both mycelium and ascospores, with a relative abundance of 66.84%. However, there was a decrease in the relative abundance of *Unclassified* from 13.27 to 9.86%, an increase in the relative abundance of *Inocybe* from 3.96 to 7.49%, a decrease in the relative abundance of *Fusarium* from 8.13 to 2.09%, and a decrease in the relative abundance of *Hortiboletus* from 7.48 to 4.39%. In conclusion, the endophytic microbial community of *Pholiota nameko* exhibited succession over time (Figure 3).

**Figure 4:**
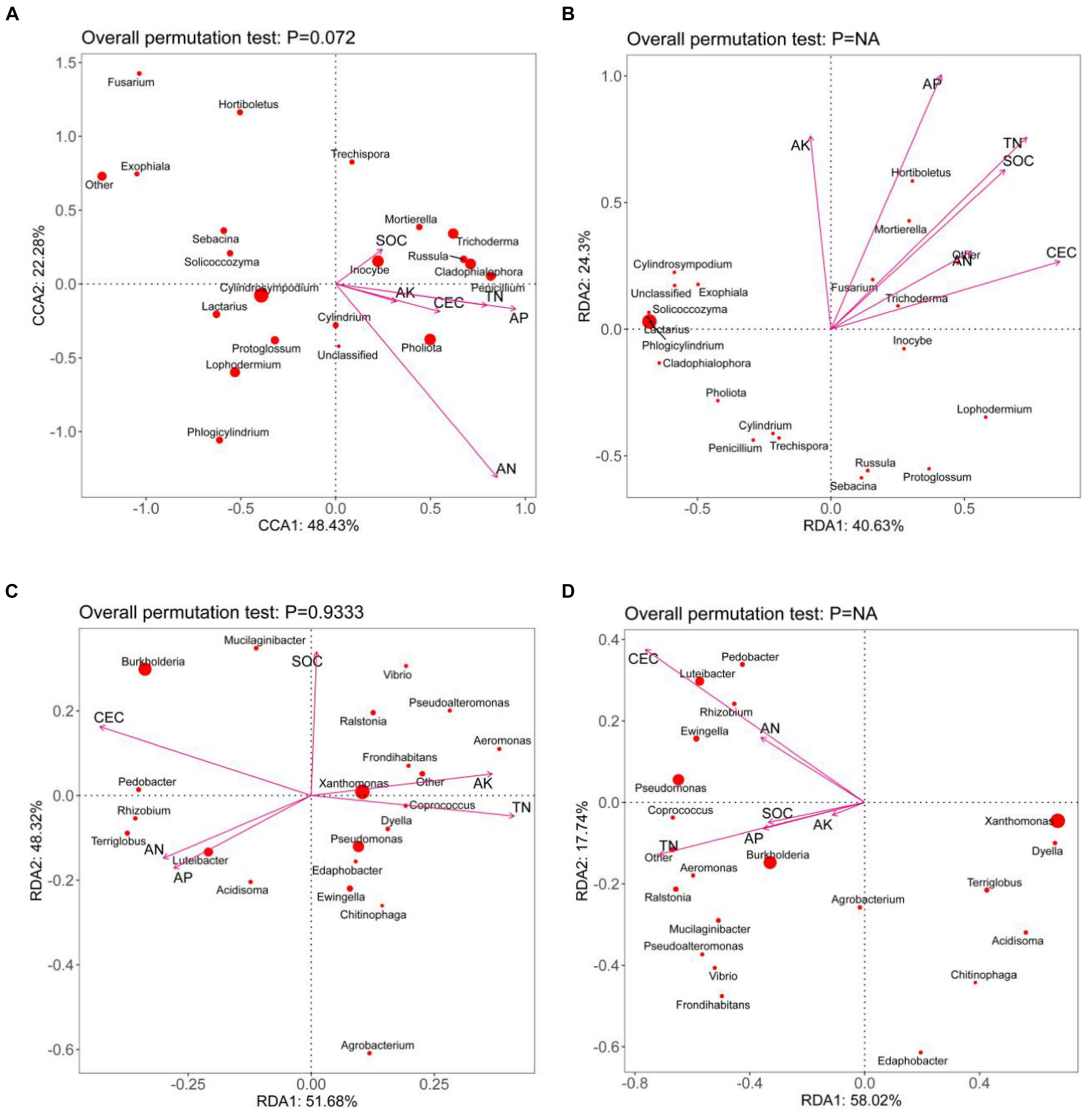
CCA/RDA analysis of the microbial community and substrate chemistry of *Pholiota nameko* medium substrate [**(A)**: *Pholiota nameko* endophytic fungal mycelium; **(B)**: *Pholiota nameko* endophytic fungal substrate; *Pholiota nameko* endophytic bacterial mycelium; slippery mushroom endophytic bacterial substrate. rDA is based on a linear model, while CCA is based on a single-peak model, the arrows in the figure represent the different environmental factors. The longer the ray, the greater the influence of the environmental factor. The angle between the environmental factors is acute, which indicates a positive correlation between the two environmental factors, and obtuse, which indicates a negative correlation between the two environmental factors].

During the *Pholiota nameko* endophytic fungal mycelium stage, the first sorting axis explained 48.43% of the variation, while the second sorting axis explained 22.28%. The strongest correlation was observed between the AN and AP contents in the culture substrate and the structure of the mycelial fungal community. The CCA analysis of the microbial community in the culture medium substrate, along with its chemical properties, accounted for a total explanation rate of 70.71% at this stage. Moving to the endophytic fungal substrate stage, the RDA analysis of the microbial community in the culture medium substrate and its chemical properties yielded a total explanation rate of 64.93%. The first sorting axis explained 40.63% of the variation, and the second explained 24.30%. At this stage, a significant correlation was found between the content of AP, TN, AK, SOC, and CEC, and the structure of the substrate fungal community, indicating a closer relationship between the microbial population in the culture medium substrate and the environmental conditions. For the stage of *Pholiota nameko* endophytic bacterial mycelium, the RDA analysis of the microbial community and chemical properties in the medium matrix showed a total explanation rate of 100%. The first sorting axis accounted for 51.68%, and the second accounted for 48.32% of the variation. The content of CEC, SOC, AN, AP, AK, and TN in the medium matrix exhibited a strong correlation with the structure of the bacterial mycelial community. Similarly, at the PN endophytic bacterial substrate stage, there was a higher correlation between CEC, TN content, and the bacterial community structure in the substrate. The RDA analysis of the microbial community and chemical properties in the medium substrate explained 75.76% of the variation. The first sorting axis accounted for 58.02%, and the second sorting axis accounted for 17.74%. Additionally, it was observed that during the development of PN from mycelium to daughter, there was a decreasing association between the concentration of SOC, AN, AP, and AK and the organization of the bacterial population (Figure 4).

### Isolation and identification of endophytic bacteria

We isolated five endophytic strains from Pholiota nameko, belonging to 5 phyla, 16 orders, 27 orders, 48 families, and 78 genera, including strains such as *Pseudomonas brassicacearum*, *Pseudomonas putida*, an uncultured *bacterium clone*, *Pseudomonas lini*, *Dyella choica*, *Dyella telluris*, *Escherichia* spp., *Salmonella* spp., *Serratia* spp., *Aspergillus* spp., *Tatum* spp., *Alicyclobacillus*, *Paenibacillus*, *Halobacillus*, *Brevibacillus*, *Aneurinibacillus*, *Virgibacillus*, *Pseudomonas aeruginosa*, *Pseudomonas fluorescens*, *Acidithiobacillaceae*, *Xanthomonas*, *Actinobacillus*, *Suttonella*, *Methylomonas*, *Alcanivorax*, *Halomonadaceae*, *Pseudomonas syringae*, and *Photobacterium*. Notably, we observed a predominance of the genus Pseudomonas among these strains.

**Figure 5:**
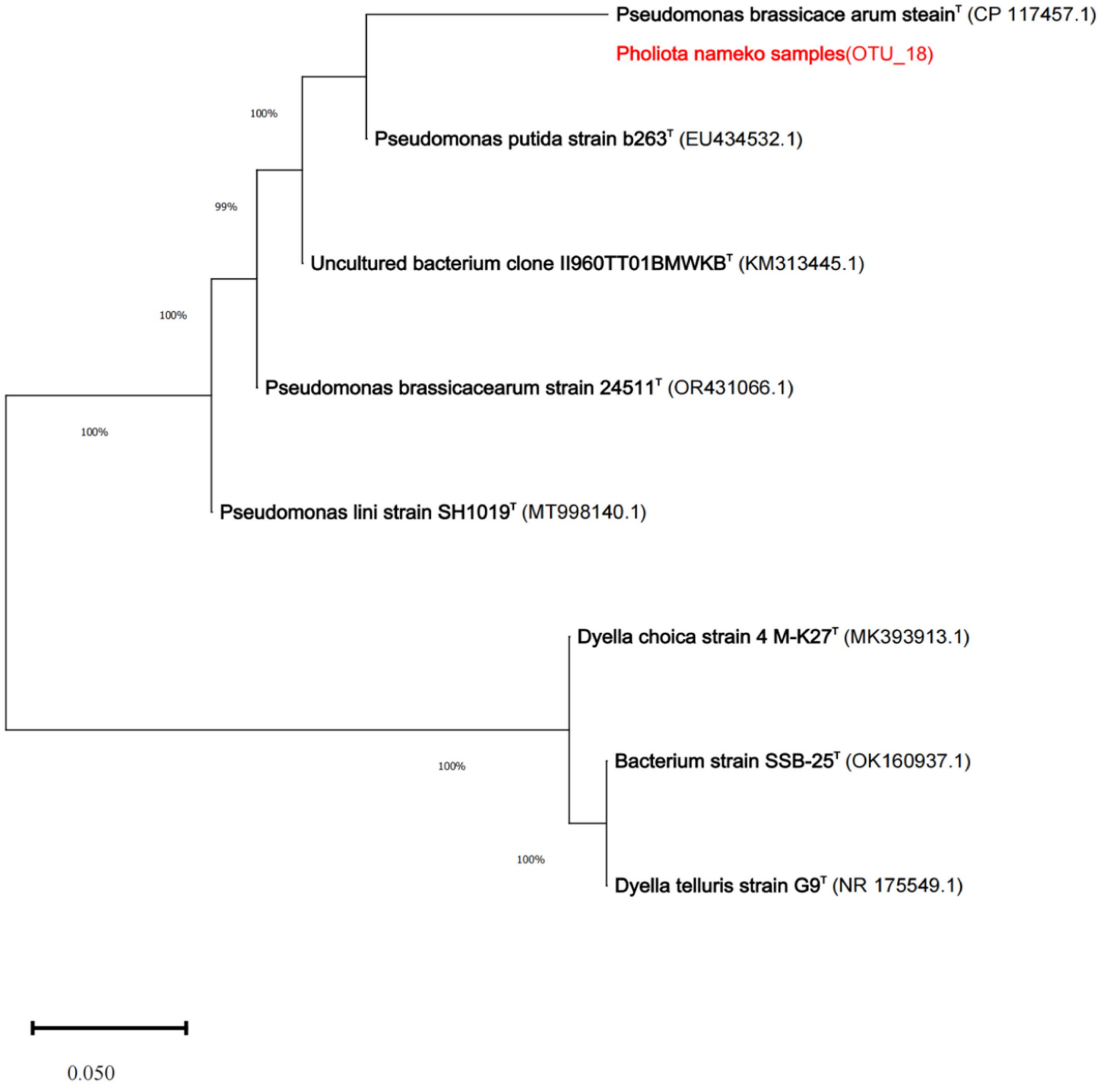
Phylogenetic tree of *Pholiota nameko* dominant genera.

The developmental tree depicted the phylogenetic relationship between the strain and several *Pseudomonas* spp. strains. It was observed that the *Pseudomonas putida strain* (EU434532.1) exhibited the highest homology with the *Pholiota nameko* isolate, with a similarity of 99.73%. Consequently, it could be confidently identified as a member of *Pseudomonas* spp. genus. Furthermore, through 16SrDNA identification, it was determined that the isolated strains predominantly belonged to the *Pseudomonas* spp. species genus, with *Pseudomonas* being the dominant strain. Hence, the endophyte of *Pholiota nameko* was identified as *the Pseudomonas brassicacearum strain* (Figure 5).

### Effect of *pseudomonas* BAS on the autolysis phenomenon of the *Pholiota nameko*

**Figure 6:**
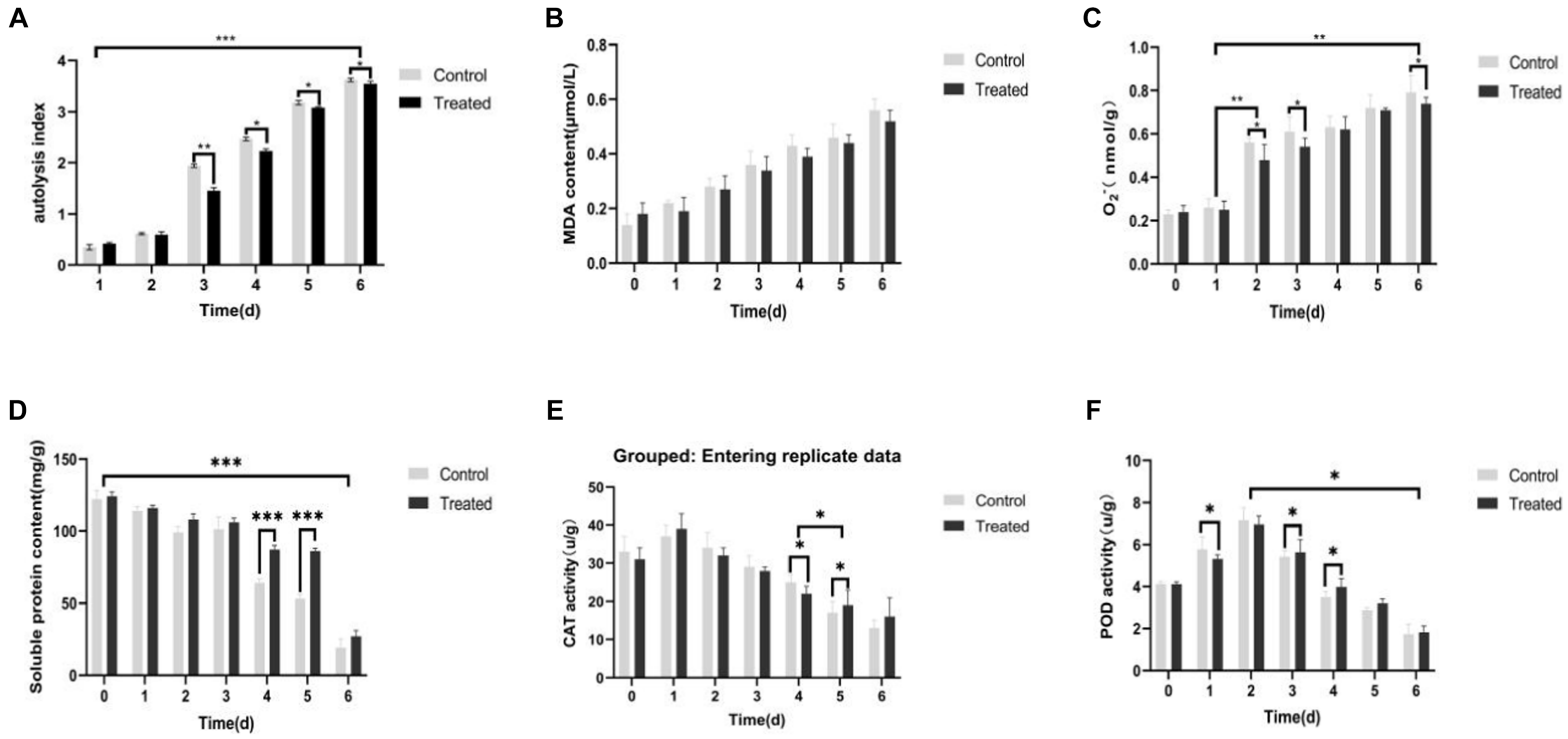
Effect of *Pseudomonas* BAS on the autolysis phenomenon of *Pholiota nameko*. **(A)** Effect of *Pseudomonas* BAS on autolysis index of *Pholiota nameko*; **(B)** effect of *Pseudomonas* BAS on MDA content of *Pholiota nameko*; **(C)** effect of *Pseudomonas* BAS on O2-content of *Pholiota nameko*; **(D)** effect of *Pseudomonas* BAS on the soluble protein content of *Pholiota nameko*; **(E)** effect of *Pseudomonas* BAS on CAT activity of *Pholiota nameko*; **(F)** effect of *Pseudomonas* BAS on POD activity of *Pholiota nameko*. *p < 0.05, **p < 0.01, and ***p < 0.001.

When *Pholiota nameko* was coated with *Pseudomonas BAS* and incubated at room temperature with sterile water as the control, the *Pholiota nameko* substrates exhibited slight expansion of the cap, and the autolysis index increased from 0 to 0.5 within 1–2 days. On the other hand, the *Pholiota nameko* coated with *Pseudomonas BAS* experienced a faster rate of autolysis throughout the 3 days, while the autolysis rate of the control group decreased over the same period. The most rapid autolysis rate was observed in the *Pholiota nameko* at (Kawamura et al., 2007; Abreu et al., 2021; Shao et al., 2022; Zhang et al., 2022) days, with a sharp increase from 0.8 to 3.5. By day 6, the majority of the *Pholiota nameko* had undergone autolysis and decomposed, as evidenced by the presence of black sap, reflected by an autolysis index of 3.7. The presence of *Pseudomonas BAS* appeared to inhibit the autolysis of *Pholiota nameko* as the autolysis index in the control group consistently exceeded that of the *Pseudomonas BAS*-coated *Pholiota nameko* (Figure 6A). MDA, a byproduct of lipid peroxidation in cell membranes, is a primary indicator of autolytic destruction in edible mushroom substrates such as *Pholiota nameko*. Excessive accumulation of MDA can damage organelles and plasma membranes, accelerating autolysis. Although the MDA content in the *Pseudomonas* BAS-treated *Pholiota nameko* was marginally lower than that in the control group, the difference was not statistically significant. Both the control group and the *Pseudomonas* BAS-treated *Pholiota nameko* exhibited an increase in MDA content from 0.28 μmol/L to 0.39 μmol/L during days 2–3. Therefore, *Pseudomonas* BAS did not prevent the autolytic senescence of *Pholiota nameko* (Figure 6B).

The O_2_^−^ content in *Pholiota nameko* substrates significantly increased over time in storage. Specifically, the O_2_^−^ content of *Pseudomonas* BAS-inoculated *Pholiota nameko* substrates grew from 0.48 nmol/g-min to 0.54 nmol/g-min, while the control group exhibited an increase from 0.26 nmol/g-min to 0.56 nmol/g-min. The rise in O_2_^−^ content was particularly noticeable between day 1 and day 2 as well as between day 4 and day 6. Between these time ranges, the O_2_^−^ content of *Pholiota nameko* substrates inoculated with *Pseudomonas BAS* reached 0.96 nmol/g-min, surpassing the control group (0.79 nmol/g-min). This indicates that *Pseudomonas BAS* infection partially hinders the ability of *Pholiota nameko* to produce O_2_^−^ during its metabolic process, thus reducing the damage caused to macromolecules in the *Pholiota nameko*’s body (Figure 6C).

The accelerated period of autolysis occurred between day 3 and day 4, resulting in significant changes in the protein content. Specifically, the soluble protein content of *Pholiota nameko* decreased from 97 mg/g to 68 mg/g in the control group and from 115 mg/g to 85 mg/g in the *Pseudomonas* BAS-inoculated *Pholiota nameko* substrates. The late period of autolysis, lasting from day 4 to day 6, showed a leveling off of the soluble protein content. Overall, the *Pholiota nameko* inoculated with *Pseudomonas BAS* exhibited a higher soluble protein content than the control group. This indicated that *Pseudomonas* BAS could partially delay the decomposition of soluble proteins in *Pholiota nameko*, enabling the retention of nutrients and extending the storage life of *Pholiota nameko* (Figure 6D).

The CAT activity of *Pseudomonas BAS*-inoculated *Pholiota nameko* was higher than that of control *Pholiota nameko*, and the CAT activity of *Pholiota nameko* in both groups slightly increased on the first day but gradually declined in the subsequent days, with the sharpest decline observed at approximately day 4 or 5 (Figure 6E). This suggested that *Pseudomonas BAS* effectively preserved the CAT activity of *Pholiota nameko* in a suitable manner.

POD plays a crucial role in plant stress tolerance by scavenging peroxides and H_2_O_2_. Research has shown that POD enzyme activity influences ethylene generation and senescent cell activity. In this study, the POD activity of two groups of *Pholiota nameko* exhibited an increasing trend, with the control group demonstrating slightly higher activity compared to *Pholiota nameko* vaccinated with *Pseudomonas BAS*. The POD activity in the control group reached its peak at 7.16 U/g on day 2. Interestingly, the POD activity of *Pseudomonas BAS*-inoculated *Pholiota nameko* was slightly lower at 6.95 U/g. From day 2 to day 6, both groups experienced a decline in POD activity, unlike the initial period from day 0 to day 2. Although the POD activity of *Pseudomonas BAS*-inoculated *Pholiota nameko* was marginally higher than that of the control group, the overall decrease in POD activity suggested that *Pseudomonas BAS* had a limited impact on the POD activity of *Pholiota nameko* (Figure 6F).

### Phylogeny of molybdenum cofactor synthesizing proteins within the genus *Pseudomonas putida*

The genomic evolutionary tree analysis revealed that of the 253 strains analyzed, 74.9% belonged to 14 different species and contained the Moa gene. Importantly, among all the strains, MoaA, one of the proteins involved in molybdenum cofactor synthesis, exhibited the highest abundance. This finding suggests the widespread presence of the molybdenum cofactor synthesis pathway in the dominant genus of *Pholiota nameko* and *Pseudomonas putida*. Moreover, it indicates that the molybdenum enzyme-mediated anaerobic oxidative phosphorylation reaction is a major pathway for energy metabolism in *Pseudomonas putida strains*. The ability of the molybdenum enzyme to catalyze anaerobic respiration appears to play a crucial role in the adaptation of *Pseudomonas putida strains* to extreme environments.

**Figure 7:**
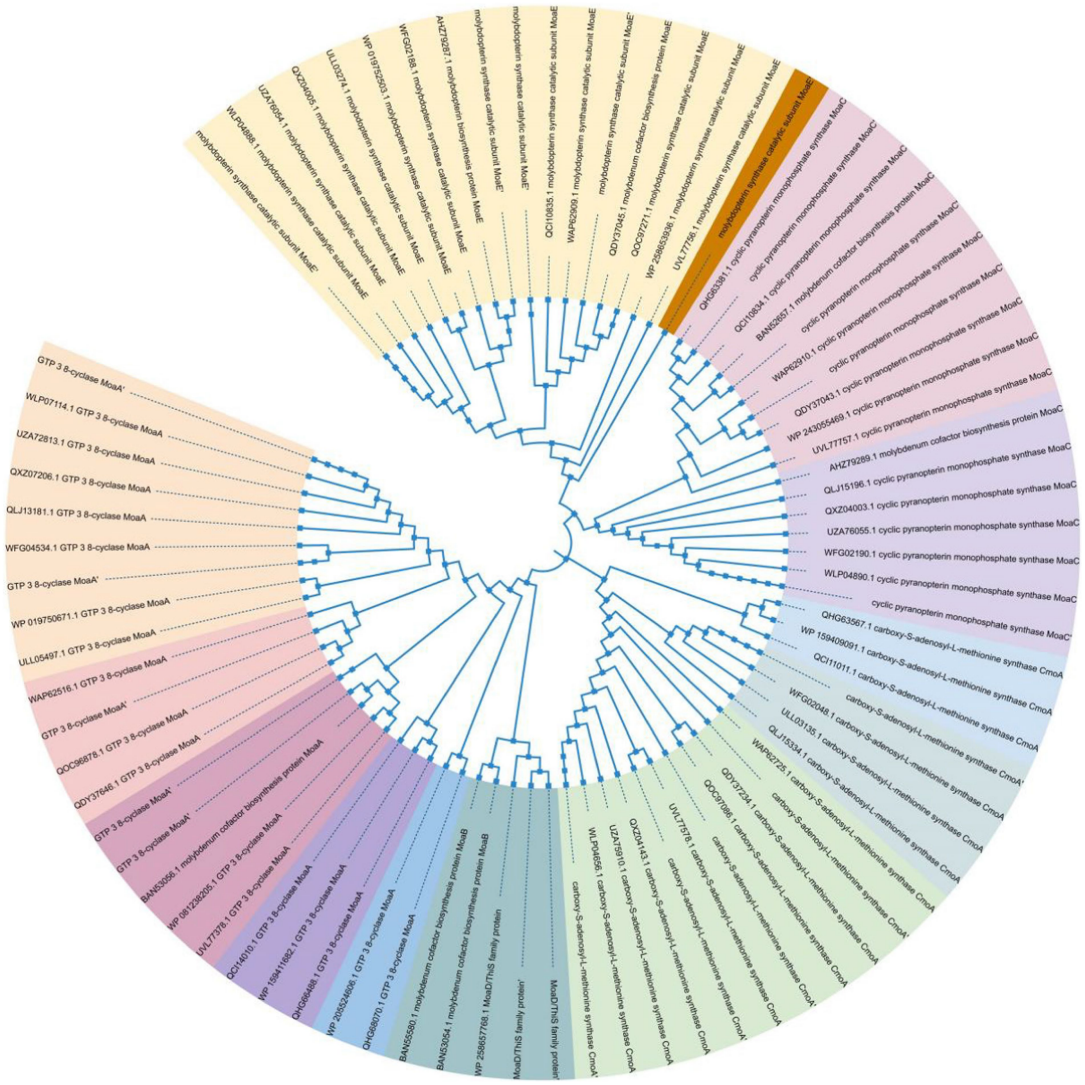
Phylogeny of MoA within the dominant genus of *Pseudomonas putida*.

Anaerobic environment-specific bacteria may have evolved additional anaerobic energy metabolic pathways to enhance their survival chances. Many bacteria possess the ability to respire in anaerobic conditions by utilizing various substrates as electron acceptors in oxidative phosphorylation. Key catalysts for this type of energy metabolic reaction are molybdenum enzymes, specifically those from the DMSOR and SO enzyme families. These enzymes enable anaerobic respiration by utilizing electron acceptors such as DMSO, TMAO, nitrate, sulfur, and even toxic metals. Moreover, molybdenum enzymes exhibit versatility in their operation, functioning effectively across a wide range of redox potentials. For instance, thiosulfate oxidases can catalyze reactions at potentials as low as −400 mV, while perchlorate reductases can remain active and catalyze reactions at potentials as high as 700 mV. This adaptability allows bacteria to survive in extremely acidic or alkaline environments. Through our research, it was discovered that the capacity of molybdenum enzymes to facilitate anaerobic respiration with a diverse range of substrates acting as electron acceptors, even within a wide pH range, was a crucial mechanism aiding *Pseudomonas putida* in adapting to various habitats, especially challenging ones (Figure 7).

## Discussion

Bioendophytes are a diverse group of microorganisms inhabiting the internal tissues of organisms, relying on host metabolites as their source of nutrients. These endophytes establish a mutually beneficial symbiotic relationship with their hosts (Pivato et al., 2017). They contribute to organism growth and development by enhancing nutrient uptake, competing with pathogenic bacteria for ecological niches, producing antimicrobial substances during metabolism, and inducing host resistance. The composition of endophytic communities varies considerably based on factors such as the ecological environment, different parts of the host plant, and the growth stage (Tiwari and Gupta, 2012). Previous research has explored the connection between organisms and endophytes using conventional tissue isolation, high-throughput sequencing, and artificial recombination of biological communities (Wang et al., 2015). In this study, we employed second-generation sequencing of the 16S rRNA gene to identify the bacterial and fungal communities associated with various developmental stages of *Pholiota nameko*. Importantly, this study is the first to investigate the composition, abundance, distribution, and temporal dynamics of endophytic bacteria in *Pholiota nameko*. Our findings reveal substantial variations in the content, number, location, and timing of endophytic bacteria within *Pholiota nameko*.

Furthermore, our analysis revealed *Pseudomonas BAS* as the predominant genus among the endophytes inhabiting *Pholiota nameko*. We proposed that *Pseudomonas BAS* played a crucial functional role in various aspects of *Pholiota nameko*’s biology. First, previous studies by Wang Zhiwei have demonstrated the growth-promoting effect of *Pseudomonas* BTa14 and Bar25 on grape and apple seedlings, attributed to their ability to enhance the production of IAA, CTK, and GA hormones (Kong and Glick, 2017). Similarly, research conducted by Zhao Yin et al. showed that endophytic fungi from Bletilla splendens produced diverse phytohormones, thereby significantly enhancing the germination of rice and oilseed seeds (Zhang et al., 2020). Furthermore, M. maplei Stempotrichum has been found to stimulate plant growth by promoting the formation of rhizomes in the roots, facilitating nitrogen uptake, augmenting the synthesis of IAA and ABA hormones, and improving microbiological and chemical soil properties through toxin degradation (Waqas et al., 2012; Zhang et al., 2020).

Second, a study conducted by Waqas et al. (Armada et al., 2014) demonstrated that endophytic fungi establish a symbiotic relationship with plants, contributing to the enhancement of stress tolerance in PN. This symbiotic association played a crucial role in improving the uptake of essential nutrients such as potassium, calcium, and magnesium under salt-stress conditions. Furthermore, this symbiotic relationship mitigated stress by reducing the activities of various enzymes, including reduced glutathione, catalase, peroxidase, and polyphenol oxidase. Additionally, it regulated the growth of the host plant by producing gibberellin and indoleacetic acid, thus promoting stress tolerance. Another research study conducted by Bressan and Figueiredo (2010) suggested that endophytic bacteria enhanced the concentration of potassium (K^+^), decreased stomatal conductance, and regulated proline accumulation. These mechanisms stimulated the growth of *Lavandula angustifolia* and enhanced the plant’s resistance to drought.

Third, Li et al. (2013) discovered that *Bacillus subtilis*, isolated from maize plants, competed with *Fusarium moniliforme Sheld* for resources within the maize plant. This competition led to a reduction in the growth rate of pathogenic bacteria and the accumulation of their toxic byproducts. Similarly, *Bacillus amyloliquefaciens*, commonly used for postharvest disease control in fruits, competes with pathogenic bacteria on the surface of fruits, thus inhibiting their colonization (Chen et al., 2016; Palmieri et al., 2020). Additionally, Matsumoto et al. (2021) found that *Rahnella aquatilis* secreted gluconic acid (Glca) in its roots, preventing acidification caused by Fusarium spinosum. This acidification prevention inhibited the growth of pathogenic fungi and interfered with their entry and colonization of plant roots. Harel et al. (2016) studied endophytic bacterial communities and observed a correlation between the distribution of *Sphingomonas* spp. members and resistance phenotypes, indicating generational heritability. Moreover, these endophytic bacteria also secrete aminomycetin, a small extracellular signaling molecule that disrupts the RpoS transcriptional cascade regulatory system, which is essential for the pathogen’s virulence. This “derived immune system” employed by the endophyte effectively inhibits disease progression.

Furthermore, through the use of 16S rDNA analysis, we identified strains co-isolated from *Pholiota nameko*. The predominant genus within the species was found to be *Pseudomonas*, with the dominant strain being identified as *Pseudomonas brassicacearum*. To investigate the impact of these strains, fresh *Pholiota nameko* was inoculated with *Pseudomonas brassicacearum*, while sterile water was used as a control by smearing it onto the *Pholiota nameko*. We assessed the autolysis of *Pholiota nameko* by calculating the autolysis index as well as by determining the content of soluble proteins, O_2_^−^ levels, MDA content, and the activities of POD and CAT enzymes in the *Pholiota nameko*. Compared to the control group, the soluble protein content in the *Pholiota nameko* inoculated with the *Pseudomonas putida strain* decreased from 125 mg/g to 85 mg/g, indicating that the strain could partially delay the decomposition of soluble proteins. Additionally, the O_2_^−^ content increased from 0.30 nmol/g/min to 0.87 nmol/g/min, suggesting that the *Pseudomonas putida strain* can inhibit the generation of superoxide anion radicals, thus reducing damage to macromolecules within the *Pholiota nameko*. However, the other investigated indices showed no significant changes compared to the control group.

The adaptability of *Pseudomonas putida strains* in harsh environments likely depends on the catalytic function of molybdenum enzymes in anaerobic respiration. Additionally, bacteria specialized for survival in anaerobic environments may evolve supplementary pathways for anaerobic energy metabolism to enhance their chances of survival. The ability of numerous bacteria to respire in anaerobic environments is closely tied to their capacity to utilize a variety of substrates as electron acceptors in oxidative phosphorylation (van Vliet et al., 2021). Anaerobic respiration in bacteria involves the utilization of diverse organic, inorganic, and toxic metals as electron acceptors. Specifically, the activity of specific molybdenum enzymes from the DMSOR and SO families acts as crucial catalysts for this energy metabolic reaction (Barrett and Kwan, 1985; Mcgrath et al., 2015; Peng et al., 2018). Our study confirmed that the ability of molybdenum enzymes to facilitate anaerobic respiration with various electron acceptor substrates and within a broad pH range was a crucial mechanism that supports the adaptive capability of *Pseudomonas putida strains* in various habitats, particularly the more challenging ones. Conversely, we observed that the distribution of proteins involved in molybdenum cofactor synthesis within the *Pseudomonas putida* genus was largely concentrated in a few species, while several strains lacked the complete synthesis pathway and possessed only one or a few related proteins. This suggests the potential presence of horizontal gene transfer contributing to the acquisition of these proteins. Further investigation is needed to determine whether other proteins exist in such strains that may serve as substitutes for the missing molybdenum cofactor-synthesizing proteins.

## Conclusion

The bacterial and fungal communities during different stages of *Pholiota nameko*’s development were profiled for the first time using Illumina second-generation sequencing of the 16S rRNA gene. Our analysis unveiled that the diversity of endophytic bacteria exceeded that of endophytic fungi within *Pholiota nameko*.

At the phylum level, *Proteobacteria*, *TM6*, *Firmicutes*, and *Bacteroidetes* were the predominant bacterial groups, while *Edaphobacter*, *Burkholderia*, and *Pseudomonas* were dominant at the genus level. Regarding endophytic fungi, *Basidiomycota*, *Ascomycota*, *Zoopagomycota*, and *Mucoromycota* were the dominant phyla, with *Pholiota*, *Inocybe*, *Fusarium*, and *Hortiboletus* being prominent genera. By analyzing strains isolated from *Pholiota nameko* using 16S rDNA, we observed that they mainly belonged to the *Pseudomonas* genus, with the *Pseudomonas putida strain* being the most abundant. Notably, our findings indicated that the *Pseudomonas putida strain* could partially delay the breakdown of soluble proteins and mitigate damage to macromolecules within *Pholiota nameko* by inhibiting the production of superoxide anion radicals. Furthermore, our results suggested that the primary energy metabolism pathway in the *Pseudomonas putida strain* was anaerobic oxidative phosphorylation. This finding implies that the synthesis pathway of the molybdenum cofactor may play a crucial role in the adaptation of *Pholiota nameko* to diverse and complex habitats.

## Data availability statement

The raw data supporting the conclusions of this article will be made available by the authors, without undue reservation.

## Author contributions

HZ: Writing – original draft. YH: Data curation, Writing – review & editing. YW: Writing – review & editing. XH: Writing – review & editing. RZ: Writing – review & editing. BL: Writing – review & editing.

## Footnotes

^1^ https://docs.qiime2.org/2019.1/

^2^ https://itol.embl.de/
