# Supplementary material for: Analysis of microbial community evolution, autolysis phenomena, and energy metabolism pathways in Pholiota nameko endophytes
Source: Front Microbiol. 2024 Apr 16;15:1319886. doi: 10.3389/fmicb.2024.1319886 (PMC11059008; doi:10.3389/fmicb.2024.1319886)
Supplement: Supplementary file 1 [file Data_Sheet_1.pdf]

Supplementary Table 1 Sequence statistics of endophytic fungi in *Pholiota nameko*

Sample name	original sequence	filtered	denoised	non-chimeric	effective sequence	effective ratio
PNM1	96373	91814	91398	90854	90854	94.3
PNM2	97881	91936	89216	89725	89725	91.7
PNM3	96502	91302	91865	90355	90355	93.6
PNS1	101676	94059	94331	94825	94825	93.3
PNS2	96613	90877	91265	91624	91624	94.8
PNS3	89369	82301	82615	82954	82954	92.8

Note: PNM stands for mycelium, PNS for substrate, and 1, 2, and 3 denote the completion of three replications, accordingly.

Supplementary Table 2 Sequence statistics of endophytic bacteria of *Pholiota nameko*

Sample name	original sequence	filtered	denoised	non-chimeric	effective sequence	effective ratio
PNM1	78062	72584	71862	72158	72158	92.4
PNM2	79088	72602	73214	73958	73958	93.5
PNM3	81211	73201	74748	75158	75158	92.5
PNS1	72332	66958	67324	67854	67854	93.8
PNS2	74648	67986	68625	68958	68958	92.4
PNS3	73134	67108	67524	67950	67950	92.9

Note: PNM stands for mycelium, PNS for substrate, and 1, 2, and 3 denote the completion of three replications, accordingly.

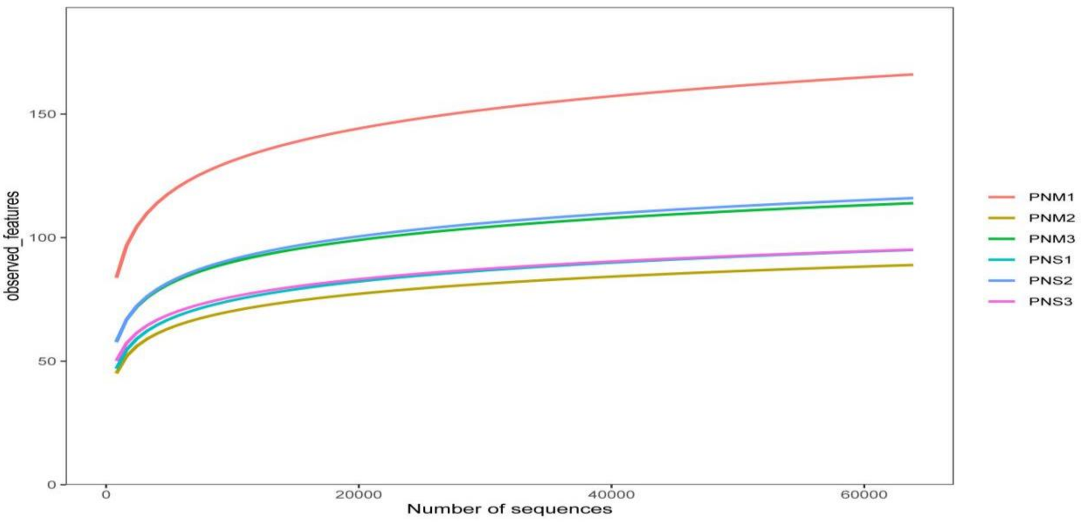

Supplementary Figure 1 Dilution curve of endophytic fungi of *Pholiota nameko*

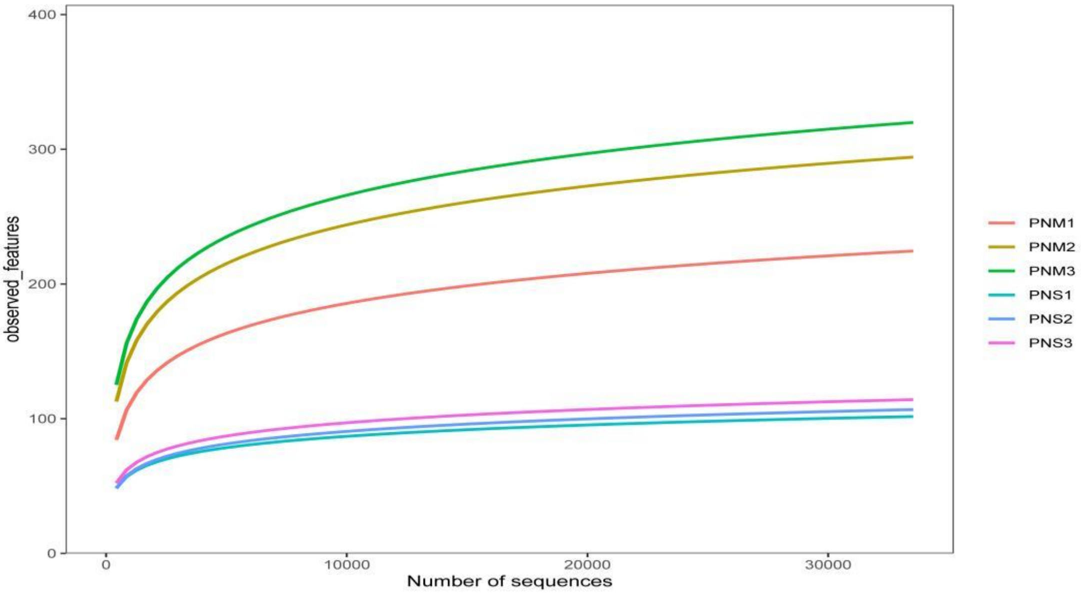

Supplementary Figure 2 Dilution curve of *Pholiota nameko* endophytic bacteria
